# Transcription regulation by CHD proteins to control plant development

**DOI:** 10.3389/fpls.2014.00223

**Published:** 2014-05-28

**Authors:** Yongfeng Hu, Yan Lai, Deyan Zhu

**Affiliations:** Jingchu University of TechnologyJingmen, China

**Keywords:** plant, CHD, chromatin, regulation, development, stress

## Abstract

Chromodomain-Helicase-DNA (CHD)-binding proteins have been characterized in various species as important transcription regulators by their chromatin remodeling activity. However, in plant the function of these proteins has hardly been analyzed before except that *Arabidopsis* PIKLE and rice CHR729 are identified to play critical roles in the regulation of series of genes involved in developmental or stress responding process. In this review we focus on how plant CHD proteins regulate gene expression and the role of these proteins in controlling plant development and stress response.

## INTRODUCTION

**FIGURE 1:**
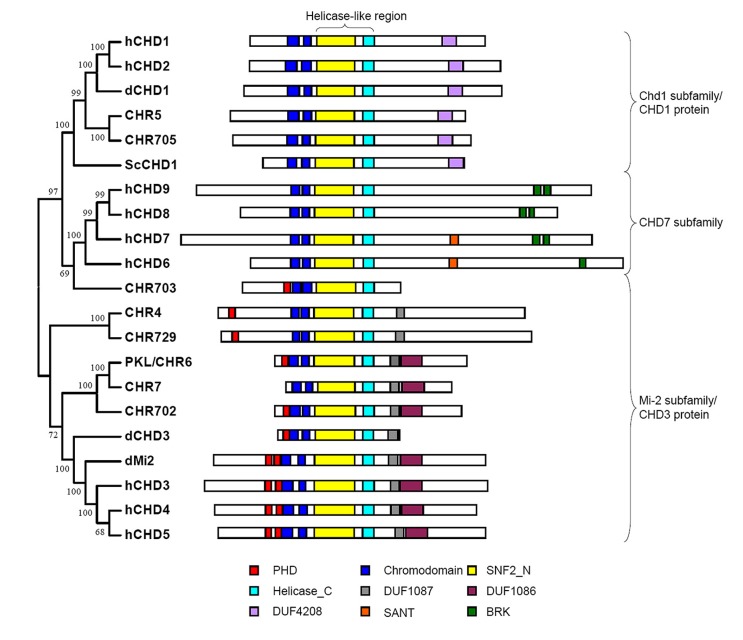
**Structural domain of CHD proteins.** Conserved domains of CHD proteins in yeast (Sc), fly (d), human (h), *Arabidopsis* (CHR4, CHR5, CHR6 and CHR7) and rice (CHR702, CHR703, CHR705 and CHR729) are represented by rectangle with different colors. The CHD proteins could be divided into three subfamilies. Each subfamily member has distinct extra domains besides double chromdomains and helicase-like region containing SNF2_N and Helicase_C domains.

Chromodomain Helicase DNA (CHD)-binding proteins, which are Snf2 family ATP-dependent chromatin remodeling factors, play important roles in the regulation of gene expression. Sequence analysis indicates that CHD proteins contain double chromodomains and Helicase-like region comprising SNF2_N and Helicase_C domains, which are critical for chromatin remodeling activity of the proteins. Based on the structure and function conservation CHD proteins could be divided into three subfamilies: Chd1 subfamily (also named CHD1 protein), Mi-2 subfamily (also named CHD3 protein), and CHD7 subfamily (Flaus et al., 2006). In addition to the common domains described above each subfamily members bear diverse extra domains (**Figure 1**). Double chromodomains of human CHD1 are associated with methylated histone H3 lysine 4 (H3K4me) which is a hallmark of active chromatin (Flanagan et al., 2005). Yeast Chd1 regulates transcription by interacting with SAGA complex which contains histone acetyltransferase and *Drosophila* CHD1 is required for histone H3.3 deposition demonstrating that the proteins in Chd1 subfamily are positively involved in transcription regulation (Pray-Grant et al., 2005; Konev et al., 2007). In Mi-2 subfamily, two PHD finger domains at N-terminal of human CHD4 bind to histone tails, while the double chromodomains of dMi-2 display DNA binding activity (Bouazoune et al., 2002; Mansfield et al., 2011). The effect of CHD3 protein on gene transcription seems to be complicated and will be discussed later. The proteins in CHD7 subfamily are homologs of *Drosophila* Trithorax-group protein, kismet, which are exclusively found in animals and also implicated in the regulation of gene expression in concert with other proteins (Bajpai et al., 2010).

In plants, there is little functional information about CHD proteins. Most studies concentrate on *Arabidopsis* PKL, which is a CHD3 protein. In recent years two papers reported the function of CHR729, a rice CHD3 protein (Hu et al., 2012; Zhao et al., 2012). From these studies we found that CHD proteins act as important epigenetic regulators to control series of genes expression and participate in the processes of development and stress response.

## PLANT CHD PROTEINS

Phylogenetic analysis of Snf2 family proteins in *Arabidopsis* and rice identified two subfamilies of CHD proteins in plant: Chd1 subfamily and Mi-2 subfamily (Hu et al., 2013). *Arabidopsis* MORPHEUS’ MOLECULE1 (MOM1) and its homologs in rice evolved from CHD3 chromatin remodelers were not considered to be CHD protein as they do not have conserved Helicase_C domain in the helicase-like region (Hu et al., 2013). Only one protein was found in Chd1 subfamily in both *Arabidopsis* (CHR5) and rice (CHR705; Hu et al., 2013). The function of these proteins has not been analyzed yet. Sequence analysis of plant CHD1 proteins indicates that they have conserved tandem chromodomain but lack the aromatic residues responsible for recognition of methylated H3 tail (Hu et al., 2013). However, the possibility could not be excluded that plant CHD1 binds to methylated H3 via the other residues. There are three members of Mi-2 subfamily in *Arabidopsis* (PKL, CHR4, and CHR7) and rice (CHR702, CHR729, and CHR703). All of these proteins contain single PHD domain and two chromodomains except *Arabidopsis* CHR7 which lacks PHD domain at N-terminal part of the protein (**Figure 1**). Phylogenetic analysis using helicase-like region showed that CHR7 was the close homolog of PKL (Hu et al., 2013). Functional analysis suggests that they act redundantly to regulate gene expression and control plant development, giving rise to the question whether PHD domain is vital for the function of PKL (Aichinger et al., 2009). Although containing both PHD domain and chromodomain rice CHR703 seems to be less homologous to the other CHD proteins and the homolog of the protein in *Arabidopsis* does not exist demonstrating that it might be evolved after the divergence of monocot and dicot (Hu et al., 2013).

## REGULATORY FUNCTION OF CHD3 PROTEIN IN PLANT

**FIGURE 2:**
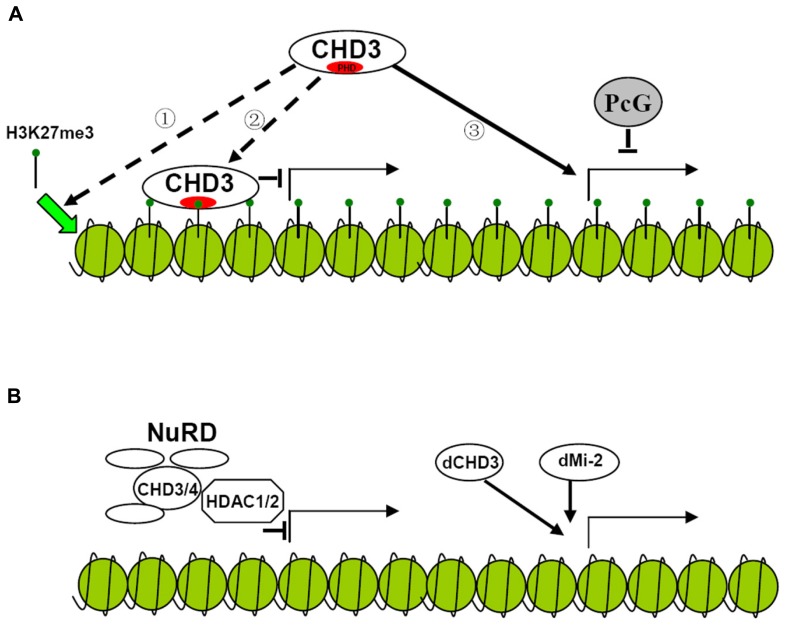
**Possible models for the regulation of gene transcription by CHD3 protein in plant and animal. (A)** Hypothesized model for the regulation of gene transcription by plant CHD3 proteins. Plant CHD3 proteins act as monomer to repress or activate gene expression. The repression involves the association with H3K27me3 by PHD domain. However, it is not known whether the binding of CHD3 protein to H3K27me3 is necessary for the deposition of the mark ① or serves as a recognition mechanism for the protein to repress target gene expression ②. Plant CHD3 proteins are also required for the expression of many PcG target genes ③. **(B)** Model for the regulation of gene transcription by animal CHD3 proteins. Animal CHD3 proteins repress gene expression by composing NuRD complex with Histone deacetylase and other subunits. In *Drosophila* dMi-2 and dCHD3 also localize to actively transcribed regions to participate in the process of transcription.

It has long been found that dMi-2 is involved in the repression of hox genes. The NuRD (Nucleosome Remodeling Deacetylase) complex which contains CHD3 protein exhibits histone deacetylase activity further improving the repressive function of the protein (Kehle et al., 1998; Xue et al., 1998; **Figure 2**). However, later results show that dCHD3 which is homologous to dMi-2 but lacks one PHD domain is associated with actively transcribed sites as well as dMi-2 (Murawska et al., 2008; **Figures 1** and **2**). This indicates that CHD3 protein may also positively participate in the process of transcription of series of genes.

In plant, there are also controversial arguments on the function of PKL. It was initially characterized to repress embryonic identity related genes and later was found to negatively regulate several genes expression. The repression mediated by PKL was found to involve trimethylation of histone H3 lysine 27 (H3K27me3), a repressive epigenetic mark. PKL target genes were enriched for H3K27me3 and mutation of PKL leads to the loss of H3K27me3 at these loci (Zhang et al., 2008, 2012). Recent study indicates that unlike animal CHD3 protein, which is a component of multisubunit complex, PKL acts as a monomer suggesting that distinct mechanism may be adopted by plant CHD3 protein to repress gene expression (Ho et al., 2013; **Figure 2**). In rice, CHR729, which is the homolog of *Arabidopsis* CHR4, was found to bind to H3K27me3 via its PHD domain *in vitro*. Loss-of-function of CHR729 results in genome-wide decrease of H3K27me3 level (Hu et al., 2012). This indicates that plant CHD3 proteins may have conserved function to repress gene expression involving H3K27me3. However, it is not clear the association of CHD3 protein with H3K27me3 is necessary for the deposition of the mark or serves as a recognition mechanism for the protein to repress target genes expression (**Figure 2**).

In contrast, the other group showed that PKL was not directly involved in the repression of the genes. Derepression of these genes and loss of H3K27me3 result from down-regulation of polycomb (PcG) genes which are activated by PKL and CHR7 synergistically (Aichinger et al., 2009). They also found that PKL was directly required for activation of several other PcG target genes demonstrating the positive role of the protein in gene transcription in plant (Aichinger et al., 2009; **Figure 2**). Since many transcription factors were down-regulated in *chr729* plants it would be interesting to test if these genes are direct targets of CHR729 to clarify if the protein also participate in the process of transcription (Hu et al., 2012). As *Arabidopsis* CHR7 which lacks PHD domain acts redundantly with PKL to activate gene expression it is plausible that PHD domain is not necessary for the activation. It is not known whether CHR7 and PKL have redundant function to repress gene expression and whether mutation of PHD domain alone would affect repressive function of the protein. Further experiments are required to confirm whether PHD domain is necessary for CHD3 protein to repress gene expression.

## PLANT CHD3 PROTEINS CONTROL MULTIPLE DEVELOPMENTAL PROCESSES AND STRESS RESPONSE

In *Arabidopsis*, PKL was found to play important roles in plant development as reviewed recently (Gentry and Hennig, 2014). It was initially reported to be involved in suppressing embryonic traits. The primary root meristem of *pkl* showed characteristics of embryonic tissue (Ogas et al., 1997). The genes specifically expressed in embryo such as *LEAFY COTYLEDON 1 *(*LEC1*), *LEAFY COTYLEDON 2* (*LEC2*), *FUSCA3* (*FUS3*) and *PHERES1* were derepressed in the mutants (Ogas et al., 1997, 1999; Dean Rider et al., 2003; Li et al., 2005). Then it was proposed that *GYMNOS*, which is the same gene as *PKL*, temporally regulated target genes expression which was also regulated by CRABS CLAW spatially to promote polarity establishment of carpel (Eshed et al., 1999). Later studies revealed that PKL may play roles in several hormones responsive genes repression. For example, repression of auxin responsive transcription activators *Auxin Response Factor 7* (*ARF7*) and *ARF9* by SOLITARY-ROOT (SLR)/IAA14 requires PKL, also named SSL2 in the study (Fukaki et al., 2006); Cytokinin-hypersensitive 2 (CKH2) which is also *PKL* encoded product act together with CKH1/AtTAF12b to regulate cytokinin responsive genes and play negative roles in cytokinin responses (Kubo and Kakimoto, 2000; Furuta et al., 2011); A subset of gibberellin-dependent responses is mediated by PKL during shoot development (Henderson et al., 2004). It was also reported that PKL control meristematic activity discrepantly in leaf and root. It acts to restrict meristematic activity in leaf but was required for maintaining root meristematic activity (Ori et al., 2000; Hay et al., 2002; Aichinger et al., 2011). Recent paper showed that transcription repressor HY5 could recruit PKL to the promoter of cell elongation genes to repress H3K27me3 at the target loci which facilitates gene transcription, demonstrating involvement of the protein in cell elongation of hypocotyl responding to light (Jing et al., 2013). However, the other CHD3 proteins in *Arabidopsis* have hardly been characterized to date. As mentioned above, *Arabidopsis* CHR7 has only been reported to suppress embryonic identity and maintain root cell identity redundantly with PKL (Aichinger et al., 2009). Although detailed analysis was not performed it has been shown that down-regulation of *CHR4* by RNAi (RNA interference) technique affects plant growth, demonstrating that this gene is also important in controlling plant development (Shaked et al., 2006).

In rice, the homolog of PKL, CHR702 seems not to be critical in developmental processes. CHR702 T-DNA insertion mutants or RNAi plants of the gene does not show any visible phenotype (Hu et al., 2012). However, mutation or down-regulation of *CHR729* affects many aspects of plant development. *chr729* plants showed short and narrow leaves, reduced stem elongation, thinner culm, and short and narrow seeds, suggesting that it might be involved in controlling organ size (Hu et al., 2012). The other study also showed that CHR729 was required for chloroplast development in adaxial leaves (Zhao et al., 2012). Function of the other CHD3 genes in rice have not been studied before. Expression analysis indicates that three genes are regulated during endosperm development especially *CHR703*, which is specifically expressed in early endosperm and down-regulated with the mature of endosperm (Hu et al., 2013). This implies that rice CHD3 proteins might be related to endosperm development.

Direct evidence that plant CHD3 proteins are involved in stress response has not been present yet. However, in *Arabidopsis* PKL was found to be necessary for the repression of *ABSCISIC ACID–INSENSITIVE3* (*ABI3*) and *ABI5* in response to ABA implicating the protein may play a role in osmoic stress (Perruc et al., 2007). PKL and CHR4 are also involved in DNA damage response as mutants or RNAi plants of the genes showed sensitive or resistant to γ-irradiation or UV-C (Shaked et al., 2006). In rice, microarray data revealed that mutation of CHR729 resulted in up-regulation of many stress-responsive genes suggesting the possible involvement of the protein in stress response (Hu et al., 2012).

## CONCLUSION AND PERSPECTIVES

Studies on *Arabidopsis* PKL and rice CHR729 have revealed that the proteins contribute to epigenetic regulation of gene expression which involves polycomb mediated H3K27me3 albeit the mechanism remains to be explored. PKL was found to control expression of several developmental genes. Despite direct targets are not clear mutants of CHR729 showed multiple developmental defects suggesting that both genes have effects on plant development. In addition, *Arabidopsis* CHD3 proteins are also involved in stress response. In light of the idea that H3K27me3 is an epigenetic mark associated with regulation of developmental and stress responsive genes it would be intriguing to disclose how CHD3 proteins and H3K27me3 are related in the matter of repressing gene expression (Zhang et al., 2007; Li et al., 2013). Plant CHD1 protein has never been analyzed before although only one protein was identified in rice and *Arabidopsis* respectively. Since distinct molecular function of CHD1 protein has been found in human, fly and yeast it is necessary to analyzed whether the protein in plant functions in the same way or adopt a novel mechanism to activate gene expression. Genetic analysis is also required to show whether plant CHD1 protein play important roles in development or stress response as CHD3 protein.

## Conflict of Interest Statement

The authors declare that the research was conducted in the absence of any commercial or financial relationships that could be construed as a potential conflict of interest.
